# Supplementary figures and images for: Flow Assisted Mutation Enrichment (FAME): A highly efficacious and efficient method to enrich Double Knockouts (DKO) after gene editing
Source: PLoS One. 2021 Mar 4;16(3):e0247375. doi: 10.1371/journal.pone.0247375 (PMC7932066; doi:10.1371/journal.pone.0247375)

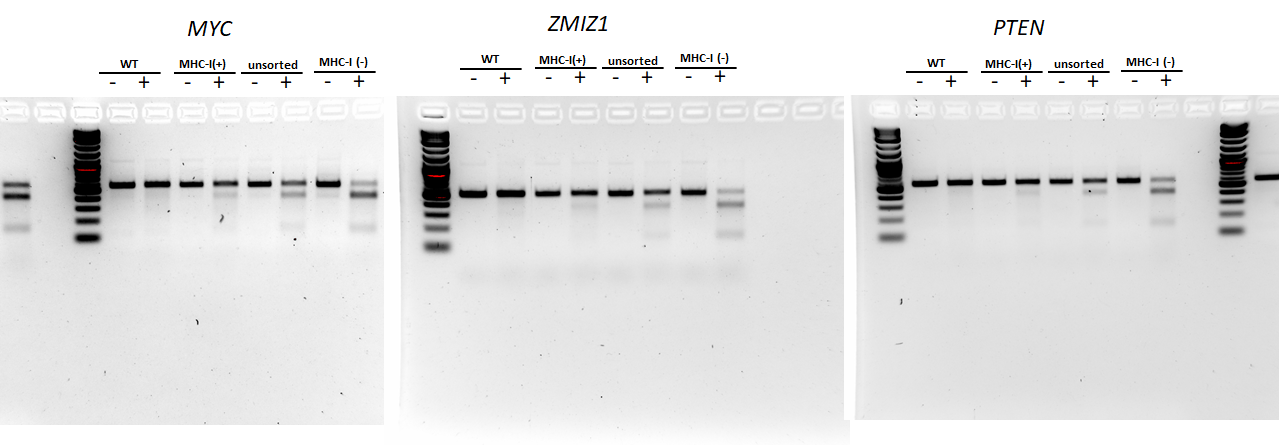

Supplement: S1 Raw image — (TIF) [file pone.0247375.s001.tif]
