# Supplementary material for: Flow Assisted Mutation Enrichment (FAME): A highly efficacious and efficient method to enrich Double Knockouts (DKO) after gene editing
Source: PLoS One. 2021 Mar 4;16(3):e0247375. doi: 10.1371/journal.pone.0247375 (PMC7932066; doi:10.1371/journal.pone.0247375)
Supplement: Attachment — Submitted filename: Comments.docx [file pone.0247375.s002.docx]

In their manuscript “Flow Assisted Mutation Enrichment (FAME): A Highly Efficacious and Efficient Method to Enriching Double Knockouts (DKO) after Gene Editing” the authors describe a new method to enrich for desired DKO based on the use of B2M as a surrogate endogenous marker gene. The concomitant disruption of B2M and target gene allows to easily identify and isolate cells in which DKO cells of both gene occurred by FACS sorting of B2M negative cells.

As the author stated in their introduction, a similar strategy has been applied with the HPRT gene, but they miss to mention that many more strategies to enrich gene edited cells has been developed over the years using transfection-selective genes and nuclease-active reporter gene .

In particular, to the latter category belongs several NHEJ-based surrogate reporter system that allows selecting by FACS or through antibiotic selection the edited cells on the basis of the cutting of the target sequence on a reporter plasmid (Kim et al, Nat Methods; Ramakrishna et al Nat. Commun; Certo et al, Nat Methods; Niccheri et al, Cell. Mol. Life Sci).

Since the cutting of two sgRNA are independent events is not necessarily granted that high efficiency of cutting at B2M locus is linked to high efficiency of cutting at other genomic sites; NHEJ-based surrogate reporter system allows instead to select cell population in which the target sgRNA is surely active.

The author should comment those other enrichment methods and discuss which are the advantage and/or cons compared to their strategy.

I also have other major and minor comment to add to the authors’ work.

Major comments:

1. The authors approach cannot be used as a universal method to enrich DKO cells. I see a major limitation in the use of B2M as surrogate reporter gene for the entire immunology field. As the author stated, since B2M has an important function for immune recognition this approach cannot be applied to select DKO clones for genes whose function is intended to be studied in the contest of immune function, i.e. cancer immunotherapy for example. Ex-vivo co-colture experiments cannot be performed with B2M negative cells without avoiding confounding effect.
2. The author also stated that B2M disruption can be useful to generate hypo-immunogenic cells with therapeutic potential but is it known that MHC-I negative cells are target of NK cells lysis activity and other strategy has been developed to avoid this (Gornalusse et al, Nat Biotech 2017). I think the authors should rephrase their concluding statement on the possible therapeutic use of their approach.
3. The authors’ work doesn't contain any statistic. How many times the experiments have been repeated for each targeted gene?

Minor comments:

1. The author should explain better what is the B2M minus cells in the WT untreated cell population. Is that a sorting artifact or those are real B2M negative cells?
2. The author should include quantification for all the NHEJ assays and also report a statistic of their experiments when applicable.
3. The title of the manuscript contains a typo. I would correct “to enriching” with “to enrich” or “for enrichment”.
